# Supplementary material for: Nuclear Factor of Activated T Cells-5 Regulates Notochord Lumenogenesis in Chordate Larval Development
Source: Int J Mol Sci. 2022 Nov 19;23(22):14407. doi: 10.3390/ijms232214407 (PMC9698811; doi:10.3390/ijms232214407)
Supplement: Supplementary file 1 [file ijms-23-14407-s001.zip › ijms-1988538-supplemental figures.pdf]

International Journal of *Molecular Sciences*

Article

Nuclear Factor of Activated T Cells-5 Regulates Notochord Lumenogenesis in Chordate Larval Development

Muchun He [1], Jiankai Wei [1,2], Yuting Li [1] and Bo Dong [1,2,3,*]

[1] Sars-Fang Centre, MoE Key Laboratory of Marine Genetics and Breeding, College of Marine Life Sciences, Ocean University of China, Qingdao 266003, China
[2] Laoshan Laboratory, Qingdao 266237, China
[3] Institute of Evolution and Marine Biodiversity, Ocean University of China, Qingdao 266003, China;
* Correspondence: bodong@ouc.edu.cn; Tel.: +86-0532-82032732

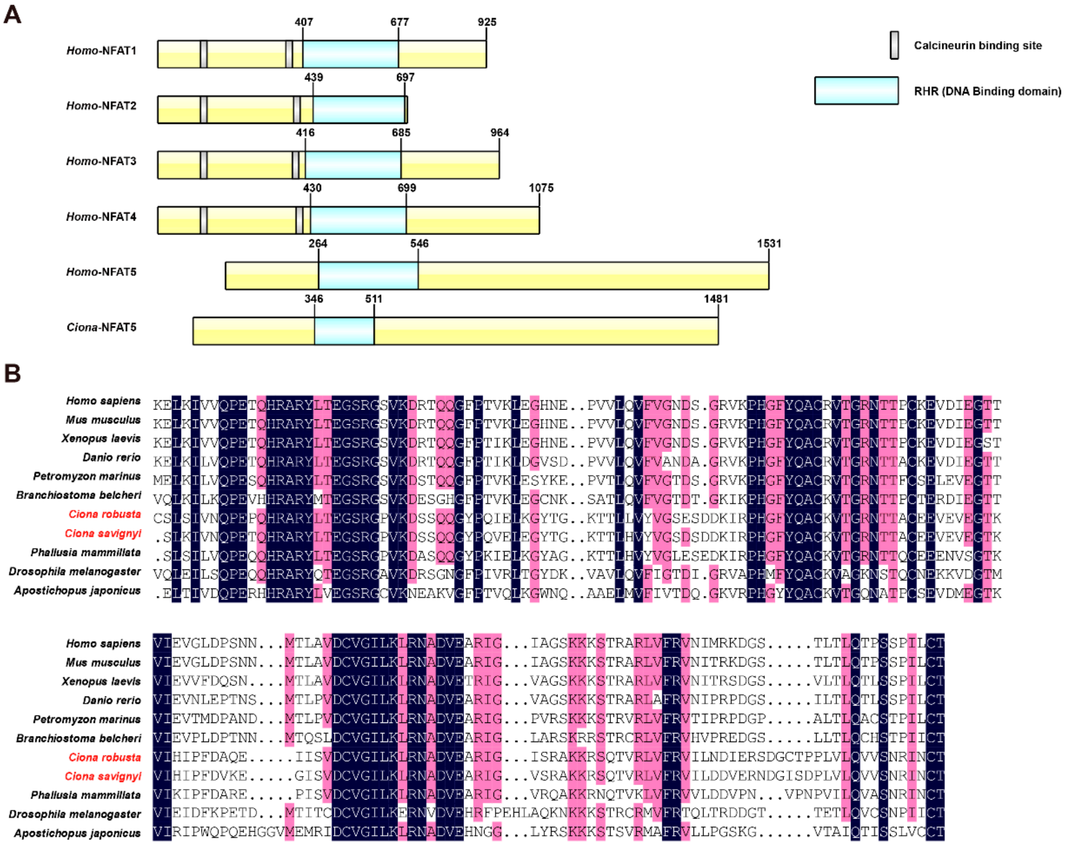

Figure S1: Schematic diagrams of the NFAT family and the sequence alignment of DNA binding domain of NFAT5. (A) Schematic diagrams of the domain organization of the NFAT family. Light blue rectangles indicate conserved RHR (DNA binding domain) and grey ones indicate calcineurin binding site. (B) The multiple DBD (DNA binding domain) sequence alignment of different species of NFAT5 sequence. The DBD sequences are highly conservative.

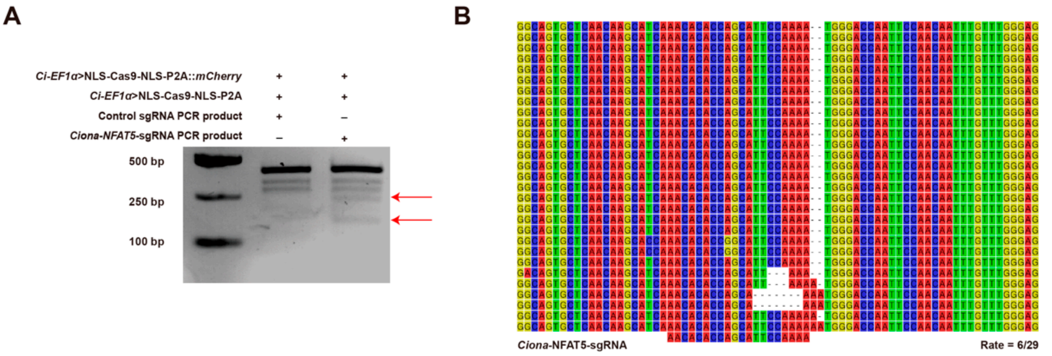

Figure S2: The knockout efficiency and targeted site of *Ciona-NFAT5* gRNA. (A) The sgRNA efficiency verification by T7 endonuclease I assay. The genomic DNA was degraded compared to control sgRNA group. The red arrow indicates the extra two bands. (B) Mutation efficiency was detected by sequencing of *Ciona-NFAT5* gRNA2. Mutation occurred in 6 of the 29 sequencing results. Mutation efficiency is 20.69 %.

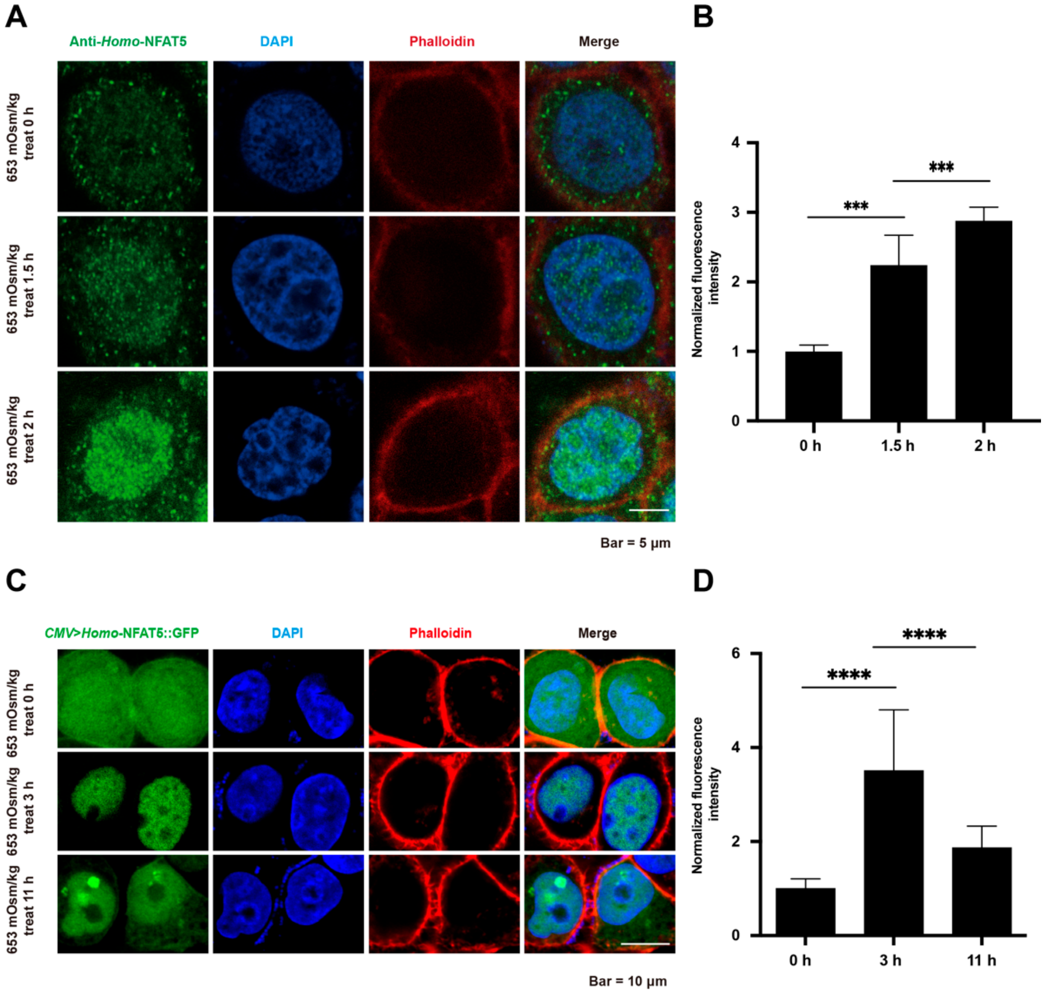

Figure S3: Endogenous and exogenous *Homo*-NFAT5 all significantly enters the nucleus after hypertonic treatment. (A) Immunohistochemical experiments of *Homo*-NFAT5. Representative cell images at 0, 1.5 and 2 h after hypertonic treatment (653 mOsm/kg). (B) Compared with the 0 h, the nucleus/cytoplasm ratio of *Homo*-NFAT5 fluorescence intensity increases significantly in hyperosmolarity group (653 mOsm/kg 1.5 h and 2 h). (C) HeLa cells were transfected with *Homo*-NFAT5. Representative cell images at 0, 3, and 11 h after hypertonic treatment (653 mOsm/kg). (D) Quantification of *Homo*-NFAT5 translocation from the cytoplasm to the nucleus upon hypertonic treatment. The fluorescence intensity of nucleus/cytoplasm was measured by image J. Scale bar represents 10 μm.

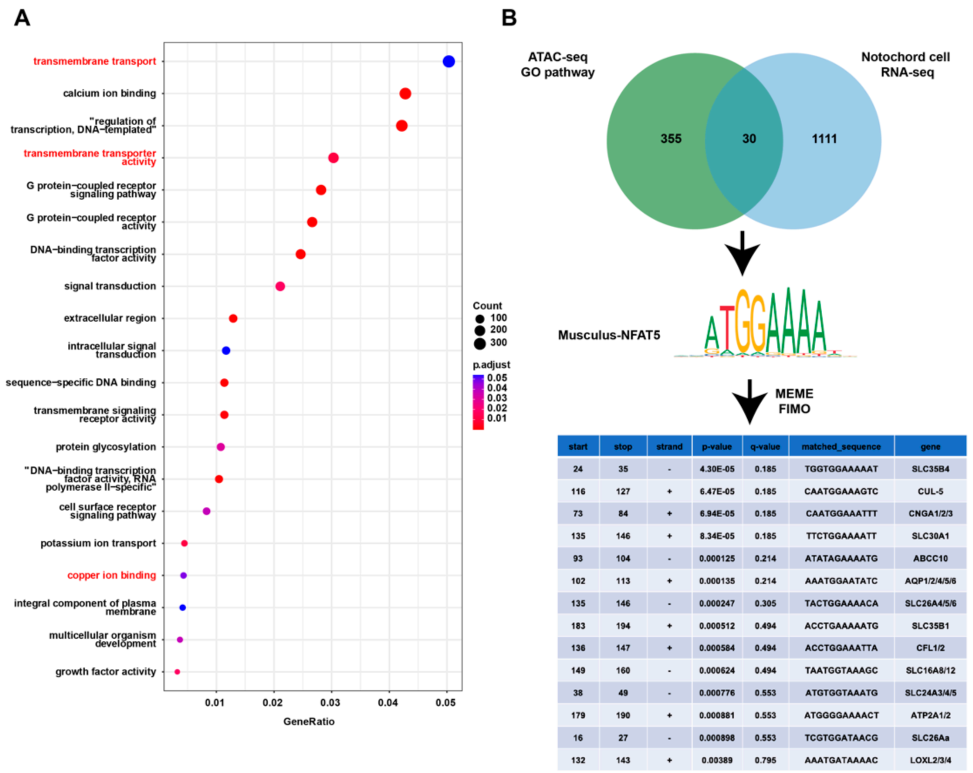

start	stop	strand	p-value	q-value	matched_sequence	gene
24	35	-	4.30E-05	0.185	TGGTGGAAAAAT	SLC35B4
116	127	+	6.47E-05	0.185	CAATGGAAAGTC	CUL-5
73	84	+	6.94E-05	0.185	CAATGGAAATTT	CNGA1/2/3
135	146	+	8.34E-05	0.185	TTCTGGAAAATT	SLC30A1
93	104	-	0.000125	0.214	ATATAGAAAATG	ABCC10
102	113	+	0.000135	0.214	AAATGGAATATC	AQP1/2/4/5/6
135	146	-	0.000247	0.305	TACTGGAAAACA	SLC26A4/5/6
183	194	+	0.000512	0.494	ACCTGAAAAATG	SLC35B1
136	147	+	0.000584	0.494	ACCTGGAAATTA	CFL1/2
149	160	-	0.000624	0.494	TAATGGTAAAGC	SLC16A8/12
38	49	-	0.000776	0.553	ATGTGGTAAATG	SLC24A3/4/5
179	190	+	0.000881	0.553	ATGGGGAAAACT	ATP2A1/2
16	27	-	0.000898	0.553	TCGTGGATAACG	SLC26Aa
132	143	+	0.00389	0.795	AAATGATAAAAC	LOXL2/3/4

Figure S4: Downstream gene screening of *Ciona-NFAT5*. (A) Top 20 GO terms enriched in genes which corresponding to the lumen expension stage (22 hpf). (B) 30 genes were identified after cross ATAC-Seq and notochord cell RNA-Seq data. Motif binding prediction (MEME software) were further applied to narrow the targeted genes.

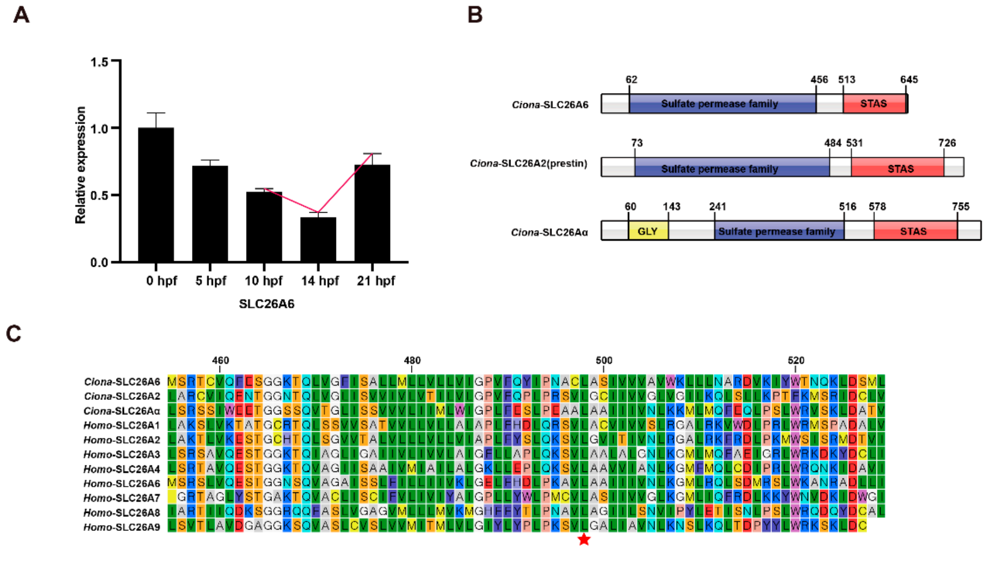

Figure S5: The mRNA expression profile of SLC26A6, the conserved domains and sequence alignment within SLC26A family proteins. (A) Expression profile of *Ciona-SLC26A6* at different developmental stages. (B) Schematic diagrams of the domain organization of *Ciona*-SLC26A6, SLC26A2 and SLC26A*α*. Deep purple indicate a sulfate transporter domain, dark red indicate STAS (a sulfate transporter and anti-sigma factor antagonist), yellow indicate GLY motif. (C) Mutant with single amino acid substitutions in regions that are highly conserved with human SLC26 proteins, *Ciona*-SLC26A6, *Ciona*-SLC26A2 and *Ciona*-SLC26A*α*. Loss of transport activity function mutant of SLC26 by substituting leucine to proline at 421 site (L421P). The red star points out Leucine.
